# Synergistic effect of plasma power and temperature on the cracking of toluene in the N_2_ based product gas

**DOI:** 10.1016/j.heliyon.2023.e14237

**Published:** 2023-03-05

**Authors:** Faisal Saleem, Asif Hussain Khoja, Rabia Sharif, Abdul Rehman, Salman Raza Naqvi, Umair Yaqub Qazi, Kui Zhang, Adam Harvey

**Affiliations:** a Chemical and Polymer Engineering Department, UET Lahore, Faisalabad Campus, Pakistan; b Fossil Fuels Laboratory, Department of Thermal Energy Engineering, U.S-Pakistan Centre for Advanced Studies in Energy (USPCAS-E), National University of Sciences & Technology (NUST), Sector H-12, Islamabad, 44000, Pakistan; c School of Chemical & Materials Engineering, National University of Sciences & Technology, 44000, Islamabad, Pakistan; d Department of Chemistry, College of Science, University of Hafr Al Batin, Kingdom of Saudi Arabia; e School of Engineering, Newcastle University, Newcastle upon Tyne, NE1 7RU, United Kingdom

**Keywords:** Tar removal, Non-thermal plasma, Lower hydrocarbons, Biomass gasification

## Abstract

In this research, a dielectric barrier discharge (DBD) reactor is used to study the cracking of the toluene into C_1_–C_6_ hydrocarbons. The combined effect of parameters such as temperature (20–400 °C) and plasma power (10–40 W) was investigated to evaluate the DBD reactor performance. The main gaseous products from the decomposition of toluene include lower hydrocarbon (C_1_–C_6_). The cracking of toluene increases with power at all temperatures (20–400 °C). On the otherhand, it decreases from 92.8 to 73.1% at 10 W, 97.2 to 80.5% at 20, 97.5 to 86.5% at 30 W, and 98.4 to 93.7% at 40 W with raising the temperature from 20 to 400 °C. Nonetheless, as the temperature and plasma input power increase, the methane yield increases. At 40 W, the maximum methane yield was 5.1%. At 10 and 20 W, the selectivity to C_2_ increases as the temperature rises up to 400 °C. At 30 and 40 W, it began to drop after 300 °C due to the formation of methane and the yield of methane increases significantly beyond this temperature.

## 1 Introduction

Biomass is being used as a renewable source of energy due to the fast depletion of fossil fuels. The carbonaceous biomass is converted to gaseous fuel through the gasification technique. In this technique, solid biomass is partially oxidized at elevated temperatures with the help of gasifying medium [1]. Different parameters (gasifying medium, nature of the biomass, operating conditions, etc.) affect the final composition of fuel/product gas [2]. The gasifier product/fuel gas also contains impurities like particulate matter ash and tar [[3], [4], [5]]. The formation of tar in the gasification system is a significant issue that is necessary to be solved. Tars in the product gas can condense in filters, engines, and heat exchangers at a lower temperature. Therefore, it creates maintenance and operational problems. Hence, tar removal is necessary for the successful use of product gas [6]

Applications of non-thermal plasma reactors have been significantly increased to crack tar, as well as pollutants [[7], [8], [9], [10], [11], [12], [13], [14], [15], [16], [17]]. A packed-bed DBD reactor was used to investigate the removal of tar compound (toluene). The type of carrier gas was shown to have a substantial impact on the removal of tar compound [18]. The removal efficiency was higher in the N_2_ atmosphere than in fuel gas, due to the presence of extra reactive species in fuel gas which have a lower reaction rate with tar molecules [18,19]. Therefore, the decomposition of toluene was not efficient in the fuel gas mixture [18]. However, In an air gasifying medium, the resultant gas is made up of N_2_, H_2_, CO, CO_2_, lower hydrocarbons, and some other by-products [2]. Due to this reason, the decomposition efficiency of tar should be increased in a synthetic product gas which consists of N_2,_ CO, H_2,_ and CO_2_ by using a DBD reactor.

In the current work, the DBD reactor’s performance was studied to crack the tar compound in synthetic product gas (CO_2_:15%.CO:15%.H_2_:20%, N_2_: balance). Several experiments were performed by changing the operating conditions like power, and temperature. When compared to other components of biomass tar, toluene was selected as a tar representative due to its simple structure, good thermal stability, and lower boiling point [20,21].

## 2 Methodology

**Fig. 1:**
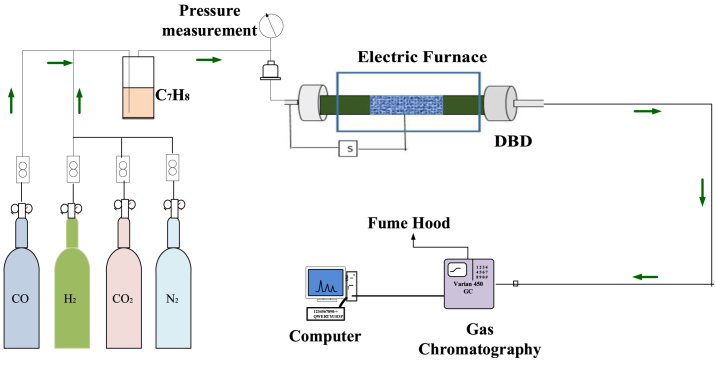
A diagram of the tar treatment experimental setup.

The experimental scheme of the setup can be seen in Fig. 1. The experimental procedure is provided in the supporting information.

## 3 Results and discussion

### 3.1 Conversion of toluene

**Fig. 2:**
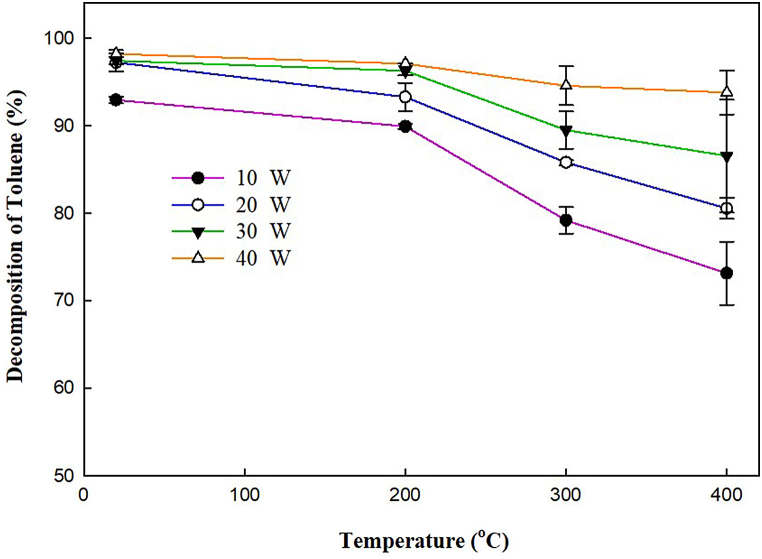
Effect of temperature on toluene conversion at various plasma input powers.

Fig. 2 shows the variations of toluene decomposition with respect to temperature and plasma input power. The furnace temperature was varied from 20 to 400 °C for each level of power. It can be noted from the graph that the decomposition of toluene decreases with increasing temperature at various levels of power. It was revealed that the cracking of toluene may decrease due to the reduction in electric insulativity of quartz at high temperatures which lowers the intensity of the discharge [22]. In a separate study, it has been reported that the toluene decomposition decreased due to the production of solid residues/soot [23]. The presence of solid residues may affect the plasma characteristics which decreases the cracking of toluene at higher temperatures. Moreover, it was revealed that the toluene decomposition decreased due to the recombination reactions of the fragments which reproduced toluene [18,24].

At all temperatures, the breakdown of toluene rises with increasing plasma input power, as seen in Fig. 2. At higher power, more high-energy electrons are produced, which contribute to the activation of reactant molecules. The electric field, electron density, and gas temperature increased with rising the plasma input power which contributed to the high cracking of toluene [[25], [26], [27]]. Moreover, the generation of reactive species (Eqs. (1), (2), (3)) due to the collision of electrons is also responsible for the high decomposition of toluene in the product gas mixture [7,28].

(1) $$N_{2}+e\;\to \;N_{2}^{*}+e$$

(2) $${CO}_{2}+e\;\to \;CO+O+e$$

(3) $$H_{2}+e\;\to \;H+H+e$$

The excited molecular states of nitrogen $N_{2}^{*}$ (N_2_ $\left(A^{3}\sum_{u}^{+}\right)$, N_2_ (B^3^∏_g_) and N_2_ (C^3^∏_u_)) produced with the impact of electrons [20,29]. It was revealed that the reaction of N_2_ $\left(A^{3}\sum_{u}^{+}\right)$ with targeted molecules is very important in mixtures containing nitrogen [30]. Previously, it has been revealed that N_2_ showed higher decomposition of toluene than CO_2_, CO, and H_2_ carrier gases [31]. The oxygen radicals which are generated through the CO_2_ decomposition, are important reactive species for the oxidative removal of toluene. Additionally, the generation of H radicals can also assist the tar compound decomposition. The energy required to break C–H bond in the methyl group of the toluene molecule is the minimum [32]. Therefore, the hydrogen abstraction from the CH_3_ could initiate the decomposition of toluene. Moreover, the C–C bond of the aromatic ring and the methyl group could be broken by the energetic electrons, producing methyl and phenyl radicals [33]. Therefore, the cracking of toluene can occur due to the following reactions (Eqs. (4), (5), (6), (7), (8)) [11,28].

(4) $${C_{7}H}_{8}+e\;\to \;{{H+C}_{7}H}_{7}+e$$

(5) $${C_{7}H}_{8}+e\;\to \;{{CH_{3}+C}_{6}H}_{5}+e$$

(6) $${C_{7}H}_{8}/Intermediate\;products+e\;\to \;LHC\;\left(C_{1}-C_{6}\right)+e$$

(7) $${C_{7}H}_{8}/Intermediate\;products+O/H/N_{2}^{*}\;\to \;\text{Products}$$

(8) $${{{C_{6}H}_{5}+C}_{7}H}_{7}\to Polymerize$$

### 3.2 Selectivity to C_2_–C_5_ hydrocarbons

**Fig. 3:**
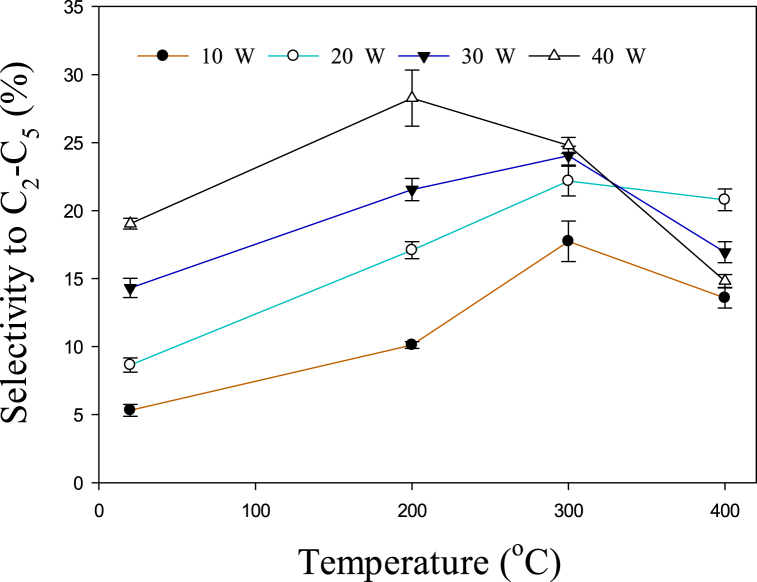
Effect of temperature on C_2_–C_5_ hydrocarbon selectivity.

**Fig. 4:**
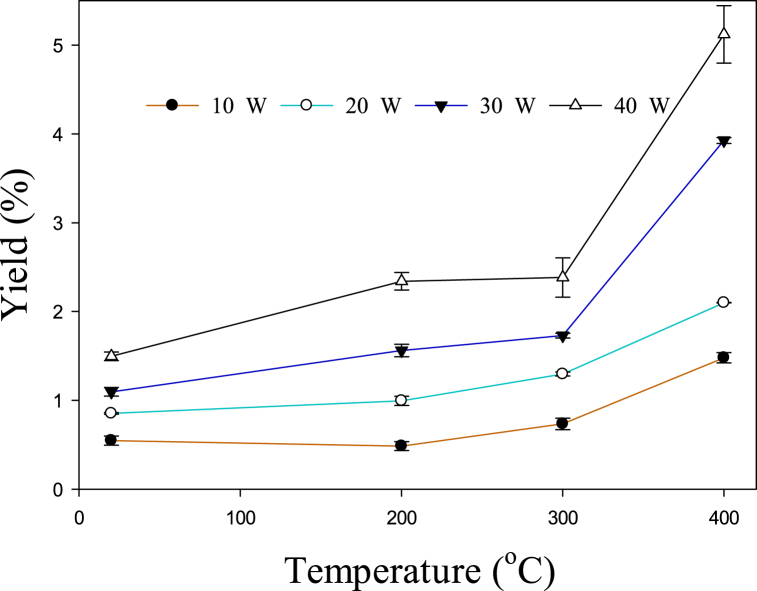
The influence of temperature on the yield of methane.

The influence of temperature and power on C_2_–C_5_ hydrocarbon selectivity is depicted in Fig. 3. It can be observed that at 10, 20, and 30 W, the selectivity of C_2_–C_5_ rises with increasing temperature from 20 to 300 °C, afterward, it started to decrease. Previously it was reported that C_2_–C_3_ hydrocarbons were present in large amounts during the conversion of toluene at 650–800 °C [34]. However, from 800 to 850 °C, the major gaseous products were C_1_–C_2_. Similarly, it has been reported that the main cracking product of toluene above 950 °C was C_1_–C_2_ hydrocarbons [35]. Therefore, the selectivity to C_2_–C_5_ decreased after 300 °C due to the formation of CH_4_. Fig. 4 shows the increase in methane output as temperature rises. . A significant rise in the methane yield can be observed after 300 °C at 30 and 40 W. In a previous study, the methane yield increased from 5.44 to 5.50% with increasing the temperature from 700 to 750 °C, and a further rise in temperature up to 850 °C increased the yield of methane to 23.75% [34]. Therefore, the overall effect of the temperature is consistent with the previous study. However, due to the presence of plasma, this similar kinds of reactions take place at lower temperatures. Toluene is converted to lower hydrocarbons at relatively lower temperatures (200–400 °C) due to the presence of active species. Fig. 3 shows that the selectivity for C_2_–C_5_ decreases even after 200 °C at 40 W. This may happen due to the abundance of reactive species at higher power. Hence, higher power lowers the conversion temperature of toluene to methane. The following recombination/dissociation reactions (Eqs. (9), (10), (11), (12), (13), (14)) may also take place to produce lower hydrocarbons [[36], [37], [38], [39]].

(9) $${CH}_{3}+{CH}_{3}\;\to \;C_{2}H_{6}$$

(10) $$C_{2}H_{6}+e\to \;{H+C}_{2}H_{5}+e$$

(11) $$C_{2}H_{5}+{CH}_{3}\to C_{3}H_{8}$$

(12) $$C_{2}H_{5}+C_{2}H_{5}\to \;C_{4}H_{10}$$

(13) $$C_{3}H_{8}+e\;\to \;{H+C}_{3}H_{7}+e$$

(14) $$C_{3}H_{7}+C_{2}H_{5}\;\to \;C_{5}H_{12}$$

### 3.3 Yield of methane

Fig. 4 shows that higher power promotes methane output. At higher power the number of reactive species increases which leads to a higher yield of the product, whereas higher temperature promotes yield due to the cracking reaction. The methane could be produced through the decomposition of toluene and the methanation reaction (Eqs. (15), (16), (17)) [18,28]. The simplest route for methane production from toluene is the combination of methyl radicals with H, which is separated from toluene as it has minimum bond dissociation energy than other C–C bonds in toluene [32]. At higher input power, the abundance of active species and energetic electrons can decompose aromatic rings as well [33,40]. These factors may also contribute to enhancing the yield of lower hydrocarbons and methane at high power and temperature.

(15) $${CH}_{3}+H\;\to \;CH_{4}$$

(16) $$\text{CO}+{3H}_{2}\;\to \;CH_{4}+H_{2}O$$

(17) $${CO}_{2}+4H_{2}\;\to \;CH_{4}+2H_{2}O$$

### 3.4 Selectivity to C_2_ and C_3_ hydrocarbons

**Fig. 5:**
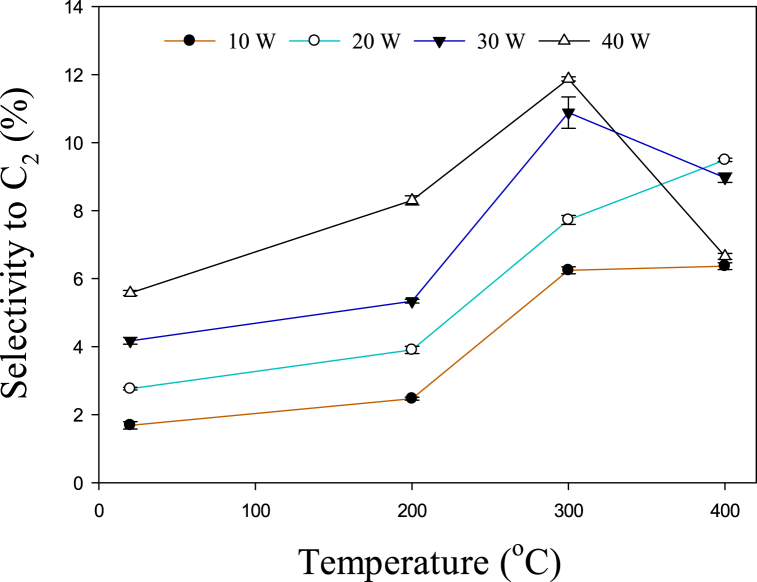
The influence of temperature on C2 hydrocarbons selectivity.

The variations in the yield of C_2_ hydrocarbons with respect to power and temperature are shown in Fig. 5. The increase in selectivity of C_2_ can be observed as the temperature rises from 20 to 400 °C, at 10 and 20 W. However, due to the existence of reactive species, it began to decline after 300 °C at 30 and 40 W. In the thermal decomposition of toluene, this behavior was observed above 800 °C, where the amount of C_2_ decreased to 42.2% from 52.1% [34]. However, due to the impact of energetic species, this trend can be observed from 300 to 400 °C at 30 and 40 W power.

**Fig. 6:**
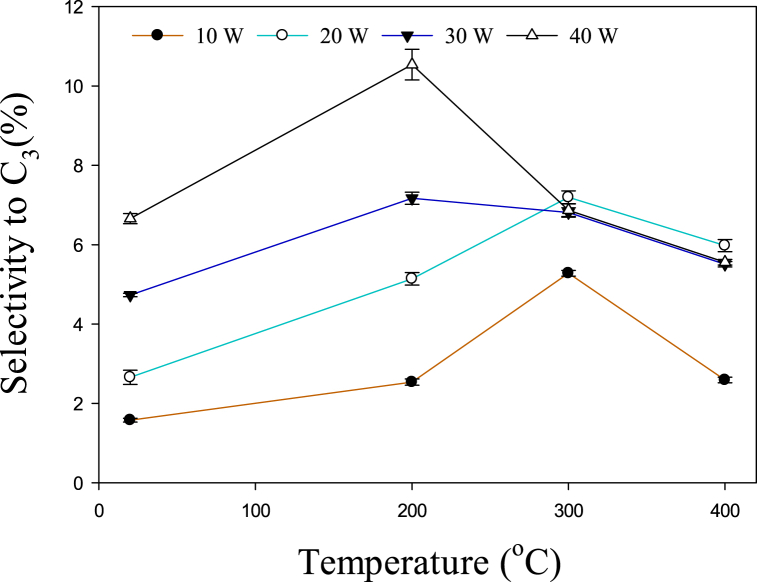
The influence of temperature on C3 hydrocarbon selectivity.

The variations of the selectivity of C_3_ with respect to temperature and power is shown in Fig. 6. At 10 and 20 W, when the temperature is raised to 300 °C, the selectivity for C_3_ increases, but then begins to decrease. Previously, it was revealed that the amount of C_3_ decreased with raising the temperature from 650 to 850 °C [34]. However, this trend is observed at relatively low temperatures from 300 to 400 °C due to the synergistic effect of temperature and plasma. At 30 and 40 W, this trend is initiated even at further lower temperatures from 200 to 400 °C. This might have occurred due to the abundance of reactive species at 30 and 40 W. Previously, it has been revealed that higher temperatures promoted the conversion of C_2_–C_4_ to methane [41]. The conversion of C_3_ to methane increased in the temperature range of 206–240 °C [41]. Hence, the combined effects of temperature and plasma increase the yield of methane significantly.

Table 1, shows the comparison of toluene decomposition with previously published research.

**Table 1:** Performance comparison of toluene decomposition with published research.

Type of Reactor	Tar model compound	Carrier gas	Flow rate (ml/min)	Plasma input Power (W)	Energy efficiency (g/kWh)	Removal efficiency (%)	References
DBD	Toluene	Mixture	40–40.6	5–40	1.9–13.2	82.6–98	[42]
DBD	Toluene	Mixture			1.95–10	63–97.7	[11]
DBD	Benzene	H_2_			2.08–8.5	48.5–97	[43]
DBD	Toluene	N_2_			1.98–15	93.4–99	[23]
DBD	Benzene	CO_2_			2.1–5	29–98.4	[43]
DBD	Toluene	H_2_			1.9–14	82–99.5	[44]
DBD	Toluene	CH_4_			1.7–3.4	21.6–86	[21]
DBD	Toluene	Mixture		10–40 W		92.8–98.4	This Study

### 3.5 Selectivity to benzene

**Fig. 7:**
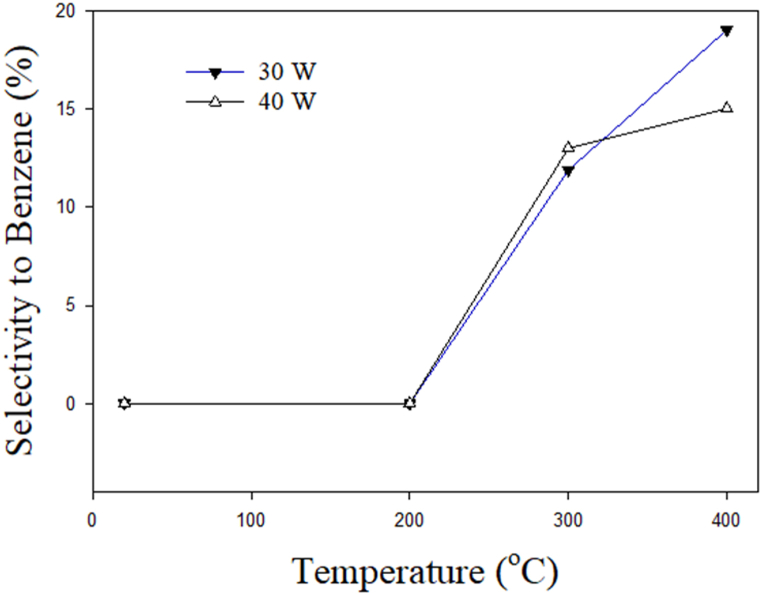
Effect of temperature on the selectivity to benzene.

Fig. 7 shows the influence of temperature on the selectivity of benzene. It can be seen that the formation of benzene does not detect up to 200 °C. However, a significant rise in the benzene selectivity can be observed as the temperature is raised from 200 to 400 °C at 30 and 40 W. The production of aromatic compounds increases as temperature rises due to the presence of H radicals [45,46]. The production of benzene may take place due to a radical substitution reaction [45]. At 40 W and 400 °C, the selectivity to benzene decreases due to the abundance of active species at higher power.

## 4 Conclusions

In this study, the synergistic effect of plasma input power (10–40 W) and temperature (20–400 °C) is studied to evaluate the DBD reactor performance. It is observed that both parameters are the key factors to study the performance of the DBD reactor. Increasing power contributed to increasing the conversion of tar compounds. The highest conversion was achieved at 40 W at all tested temperatures. However, higher temperatures at constant power decrease the decomposition of toluene. Nevertheless, increasing temperature favoured the production of C_1_–C_6_. The synergistic effect of plasma input power and temperature has a significant effect on the lighter hydrocarbons. The C_2_ selectivity increases with increasing temperature at 10 and 20 W, and decreases after 300 °C at 30 and 40 W. Similarly for C_3_, higher temperature >200 °C at 30 and 40 W decrease selectivity to C_3_ hydrocarbons and promotes the yield of CH_4_. Overall, the combined effect of temperatures and NTP significantly lowers the conversion temperature of the tar model compound to lighter gases in synthetic N_2_-based fuel product gas.

## Author contribution statement

Faisal Saleem: Performed the experiments; Wrote the Paper.

Kui zhang and Adam Harvey: Conceived and designed the experiments.

Abdul Rehman and Asif Hussain Khoja: Analyzed and interpreted the data.

Rabia Sharif and Umair Y Qazi: Contributed reagents, materials, analysis tools or data.

Salman Raza Naqvi: contributed reagents, materials, analysis tools or data; Wrote the Paper

## Funding statement

This research did not receive any specific grant from funding agencies in the public, commercial, or not-for-profit sectors.

## Data availability statement

The data that has been used is confidential.

## Declaration of interest’s statement

The authors declare no competing interests.
