# Supplementary material for: Synergistic effect of plasma power and temperature on the cracking of toluene in the N2 based product gas
Source: Heliyon. 2023 Mar 5;9(3):e14237. doi: 10.1016/j.heliyon.2023.e14237 (PMC10025038; doi:10.1016/j.heliyon.2023.e14237)
Supplement: Multimedia component 1 [file mmc1.docx]

**Supporting information**

**Synergistic effect of plasma power and temperature on the cracking of toluene in the N_2_ based product gas**

Faisal Saleem^1,*^, Asif Hussain Khoja^2^, Rabia Sharif^1^ , Abdul Rehman^1^ , Salman Raza Naqvi^3^, Umair Y Qazi^4^, Kui Zhang^5^, Adam Harvey^5^

^1^Chemical and Polymer Engineering Department, UET Lahore, Faisalabad Campus, Pakistan.

^2^Fossil Fuels Laboratory, Department of Thermal Energy Engineering, U.S-Pakistan Centre for Advanced Studies in Energy (USPCAS-E), National University of Sciences & Technology (NUST), Sector H-12 Islamabad (44000), Pakistan

^3^School of Chemical & Materials Engineering, National University of Sciences & Technology, 44000, Islamabad, Pakistan

^4^Department of Chemistry, College of Science, University of Hafr Al Batin, Kingdom of Saudi Arabia

^5^School of Engineering, Newcastle University, Newcastle upon Tyne, NE1 7RU, United Kingdom.

**Text S1 Materials and methods**

**Experimental setup**

Fig. 1 shows a diagram of the experimental setup. The dielectric barrier discharge (DBD) reactor consisted of two coaxial quartz tubes (inner and outer tube). The outer tube has an inner diameter of 15 mm, and a length of 330 mm, while the inner tube has an outer diameter of 12 mm, and a length of 130 mm. One end of the inner tube is closed to allow the flow through annular space. Two metallic electrodes of stainless steel were used, one inside the inner tube and the other outside the external tube. The length of the discharge zone can be controlled by varying the size of the external electrode (30 mm). The plasma was produced in the annular space of the coaxial cylindrical tubes. The plasma power provided to the reactor was controlled by using a variac which connected to the plasma generator. The Plasma input power was measured using an energy meter [1, 2]. The plasma generator provides power to the DBD reactor at a frequency of about 20 kHz.

**Fig. S1** Schematic diagram of the experimental setup

The flow rate of the synthetic product gas mixture (CO_2_:15%.CO:15%.H_2_:20%, N_2_: balance) was controlled by using computer-controlled mass flow controllers, connected to gas cylinders (BOC, UK). The carrier gas mixture passes through the toluene (99.8 % anhydrous, sigma-Aldrich) bubbler to saturate with desired amount of toluene. To study the thermal effect on the distribution of the products, an electric furnace was used. The DBD reactor was placed inside a furnace to control the temperature between ambient and 400 ^o^C.

The product compositions were monitored by a Varian 450-GC equipped with TCD (Thermal conductivity detector) to measure CH_4_ and H_2_, and FID (Flame ionization detector) to measure the hydrocarbons[3].

**Text S2 Definitions of Quantities**

The removal efficiency of toluene was defined as follows:

$$\text{d}_{\text{T}}\text{=}\frac{\text{moles of toluene in }\text{input}\text{ stream-moles of toluene in outlet stream}}{\text{moles of toluene in input stream}}\text{×100}$$

The following formulae were used to calculate the yield and selectivity of different products:

$$\text{CH}_{4}\text{ yield }\left( \text{\%} \right)\text{=}\frac{\text{ moles of }\text{CH}_{4}\text{ produced}}{\text{7× Moles of }\text{C}_{\text{7}}\text{H}_{\text{8}}\text{ in + moles of }\text{CO}_{\text{2}}\text{ in}\text{ +moles of CO in}}$$

$$\text{LHC selectivity} \left( \text{\%} \right)\text{=}\frac{\text{∑ (m × moles of}\text{C}_{\text{m}}\text{H}_{\text{n}})}{\text{ 7× Moles of} \text{C}_{\text{7}}\text{H}_{\text{8}}\text{ converted}}\text{×100}$$

Where m > 1

**Reference**

1. El-Shafie, M., S. Kambara, and Y. Hayakawa, *Study of the reactor temperature effect on H2 production from steam decomposition using DBD plasma.* Energy Reports, 2020. **6**: p. 45-51.

2. Harris, J., A.N. Phan, and K. Zhang, *Cold plasma catalysis as a novel approach for valorisation of untreated waste glycerol.* Green Chemistry, 2018. **20**(11): p. 2578-2587.

3. Saleem, F., K. Zhang, and A.P. Harvey, *Decomposition of benzene as a tar analogue in CO2 and H2 carrier gases, using a non-thermal plasma.* Chemical Engineering Journal, 2018.
